# 3D pCASL-perfusion in preoperative assessment of brain gliomas in large cohort of patients

**DOI:** 10.1038/s41598-022-05992-4

**Published:** 2022-02-08

**Authors:** A. I. Batalov, N. E. Zakharova, I. N. Pronin, A. Yu. Belyaev, E. L. Pogosbekyan, S. A. Goryaynov, A. E. Bykanov, A. N. Tyurina, A. M. Shevchenko, K. D. Solozhentseva, P. V. Nikitin, A. A. Potapov

**Affiliations:** Federal State Autonomous Institution N.N. Burdenko National Medical Research Center of Neurosurgery of the Ministry of Health of the Russian Federation, Moscow, Russian Federation

**Keywords:** CNS cancer, CNS cancer

## Abstract

The aim of the study was to evaluate the role of pseudocontinuous arterial spin labeling perfusion (pCASL-perfusion) in preoperative assessment of cerebral glioma grades. The study group consisted of 253 patients, aged 7–78 years with supratentorial gliomas (65 low-grade gliomas (LGG), 188 high-grade gliomas (HGG)). We used 3D pCASL-perfusion for each patient in order to calculate the tumor blood flow (TBF). We obtained maximal tumor blood flow (maxTBF) in small regions of interest (30 ± 10 mm^2^) and then normalized absolute maximum tumor blood flow (nTBF) to that of the contralateral normal-appearing white matter of the centrum semiovale. MaxTBF and nTBF values significantly differed between HGG and LGG groups (*p* < 0.001), as well as between patient groups separated by the grades (grade II vs. grade III) (*p* < 0.001). Moreover, we performed ROC-analysis which demonstrated high sensitivity and specificity in differentiating between HGG and LGG. We found significant differences for maxTBF and nTBF between grade III and IV gliomas, however, ROC-analysis showed low sensitivity and specificity. We did not observe a significant difference in TBF for astrocytomas and oligodendrogliomas. Our study demonstrates that 3D pCASL-perfusion as an effective diagnostic tool for preoperative differentiation of glioma grades.

## Introduction

Gliomas are the most common type of primary brain tumors and account for about 80% of all malignant brain neoplasms. Preoperative predicting of glioma grade is important for planning optimal treatment strategy and predicting prognosis^1,2^.

It is well-known, that advanced diagnostic modalities, for example, MR-perfusion, are more effective in establishing brain tumor grades as compared to the routine MRI^3,4^. Today Dynamic Susceptibility Contrast (DSC) method using T2/T2* weighted dynamic imaging is an MRI “gold standard” for the tumor blood flow assessing^5–7^.

Arterial spin labeling (ASL) is a non-invasive method of measuring CBF (cerebral blood flow) values; however, its ability to evaluate glioma grades remains controversial.

There are two main types of ASL which are commonly used: pulsed ASL (PASL), and the most advanced method—pseudocontinuous ASL (pCASL). Previous studies have reported that CBF imaging using PASL and pCASL is informative for assessing gliomas, as well as differentiating high- and low-grade gliomas before surgery^8–15^, and for disease prognosis^10,16,17^.

Several studies reported a high correlation between tumor blood flow values measured by ASL-perfusion and DSC-perfusion^15,18^.

It is a well-known fact that pCASL is one of the most advanced perfusion methods^19–22^. A number of recent studies were devoted to analyzing the role of pCASL-perfusion in differentiating cerebral gliomas grades^4,23–34^. Some studies found pCASL to be ineffective for glioma differentiation^4,23^, while others, on the contrary, claimed its efficacy^24–34^. They showed contradictory results regarding sensitivity and specificity, together with a great variety of the reported threshold values. This heterogeneity of the results could be partially explained by different approaches used for selecting ROI/VOI in TBF assessment and by different methods of TBF normalization.

We decided to measure TBF values using small ROIs since we assumed that this method was more sensitive for glioma grade assessment than using large ROIs. High grade gliomas have heterogeneous structure with areas of high and low malignancy and regions of the tumor which have the highest TBF tend to show a higher malignancy^14^. Large ROIs might include tumor tissue of the different grades which could affect the sensitivity of the method.

The aim of the present study was to evaluate the predictive value of pseudocontinuous ASL-perfusion (pCASL-perfusion) in the preoperative assessment of cerebral glioma grades. We used pCASL to measure maximal absolute and normalized TBF values within small ROIs in the tumor in a large sample of patients (*n* = 253). Planning this work we hypothesizied that this method would be effective to differentiate cerebral glioma grades.

## Materials and methods

The study group consisted of 253 patients with supratentorial glial tumors (118 men; 135 women; age range 7–78 years, average age 45 ± 15 years). MRI studies were performed before any surgery, radiotherapy and chemotherapy. All patients received treatment in Burdenko Neurosurgery Center, Moscow, between 2011 and 2018 (surgical resection or stereotactic biopsy). All tumors were histopathologically assessed by experienced pathomorphologysts (Table 1).

**Table 1:** Histopathological diagnosis in patients of the studied group (WHO, 2016).

Histopathological diagnosis	Grade, WHO	Number of patients
Ganglioglioma	I	4
Papillary glioneuronal tumor	I	1
Pilocytic astrocytoma	I	5
Gemistocytic astrocytoma	II	1
Diffuse astrocytoma	II	39
Oligodendroglioma	II	14
Pleomorphic xantoastrocytoma	II	1
Anaplastic astrocytoma	III	44
Anaplastic oligodendroglioma	III	22
Anaplastic pleomorphic xantoastrocytoma	III	2
Glioblastoma	IV	118
Gliosarcoma	IV	2

65 of 253 patients had low-grade gliomas (grade I–II), and 188 had high-grade gliomas (grade III—68; grade IV—120).

MRI studies were performed using 3 T MRI scanner GE Signa HD with 8-channel head coil. Imaging protocol included axial T2 weighted images with 5 mm slice thickness and 1 mm gap; T2-FLAIR with 5 mm slice thickness and 1 mm gap; DWI ASSET with 5 mm slice thickness and 1 mm gap; axial T1 FSPGR BRAVO, isotropic voxel 1 × 1x1mm, no gap (or axial T1 SE with 5 mm slice thickness; 1 mm gap). 3D pCASL: 3D FSE, 8-way spiral whole brain scanning with following reformation for 4 mm slice thickness; FOV = 240 × 240 mm; matrix 128 × 128, ZIP 512; TR—4717 мs; TE—9.8 мs; NEX = 3; post-labeling delay (PLD)—1525 ms; pixel bandwidth—976.6 Hz/pixel. Labeling duration = 1500 ms; background suppression is applied; we did not save M0 scans for the patients but we assessed M0 homogeneity before ROI placement. Scan duration 4 min 30 s. Finally contrast-enhanced T1 FSPGR BRAVO, isotropic voxel 1 × 1 × 1mm; no gap, was performed (or axial T1 SE with 5 mm slice thickness; 1 mm gap in axial, sagittal and coronal planes).

Data processing was performed using ReadyView software (GE Healthcare). To assess tumor blood flow (TBF) values, an experienced neuroradiologist (> 5 years of experience), manually placed 4–6 ROIs on TBF color maps in the areas with absolute maximum TBF in tumor, in every slice which contained tumor. Inside every ROI mean TBF was calculated. Then one ROI (from many ROIs) with the maximum mean TBF (maxTBF) was chosen. Overall, ROIs with the area of 30 mm^2^ ± 10 mm^2^ were placed. TBF maps and postcontrast T1 or T2-FLAIR images were fused to exclude large vessels, necrosis, haemorrhages, and to assure that ROIs are placed within tumor. To assess TBF within gliomas grade II (tumors without obvious regions with TBF elevation) our expert manually placed 4–6 ROIs in tumor in every slice which contained tumor and selected one ROI with the highest TBF.

Normalized TBF values (nTBF) were obtained by normalizing absolute maxTBF to the contralateral normal-appearing white matter of the centrum semiovale (we put similar size ROI).

Statistical analysis was performed with R-project program (https://www.r-project.org), pROC library was used for ROC-analysis. We used nonparametric methods in this study. Between-group comparisons were performed using the two-tailed Mann–Whitney rank-sum tests; continuous dependences were evaluated using Spearman rank correlations.

### Study results

Maximum absolute TBF levels and normalized TBF levels are summarized in Table 2.

**Table 2:** Maximum absolute and normalized TBF values in gliomas of different grades.

Grade WHO	Mean maxTBF, ml/100 g/min	Standard deviation, ml/100 g/min	Mean nTBF	Standard deviation
I	36.5	15.5	2.3	1
II	30.8	14.2	1.7	0.7
III	122.9	85.1	6.8	4.5
IV	171.1	93.3	9.5	5.5
I + II	31.7	14.5	1.8	0.8
III + IV	153.6	93.1	8.5	5.3

We found a significant difference (*p* < 0.001) comparing maxTBF between LGG (grade I + II) and HGG (grade III + IV) groups, with higher maxTBF values in HGG patients. Tumor grades significantly correlated with both maxTBF and nTBF (Spearman’s correlation coefficient for maxTBF: 0.7 (*p* < 0.001), CI 95% (0.59–0.79) and for nTBF: 0.68 (*p* < 0.001), 95% (0.56–0.78)).

**Figure 1:**
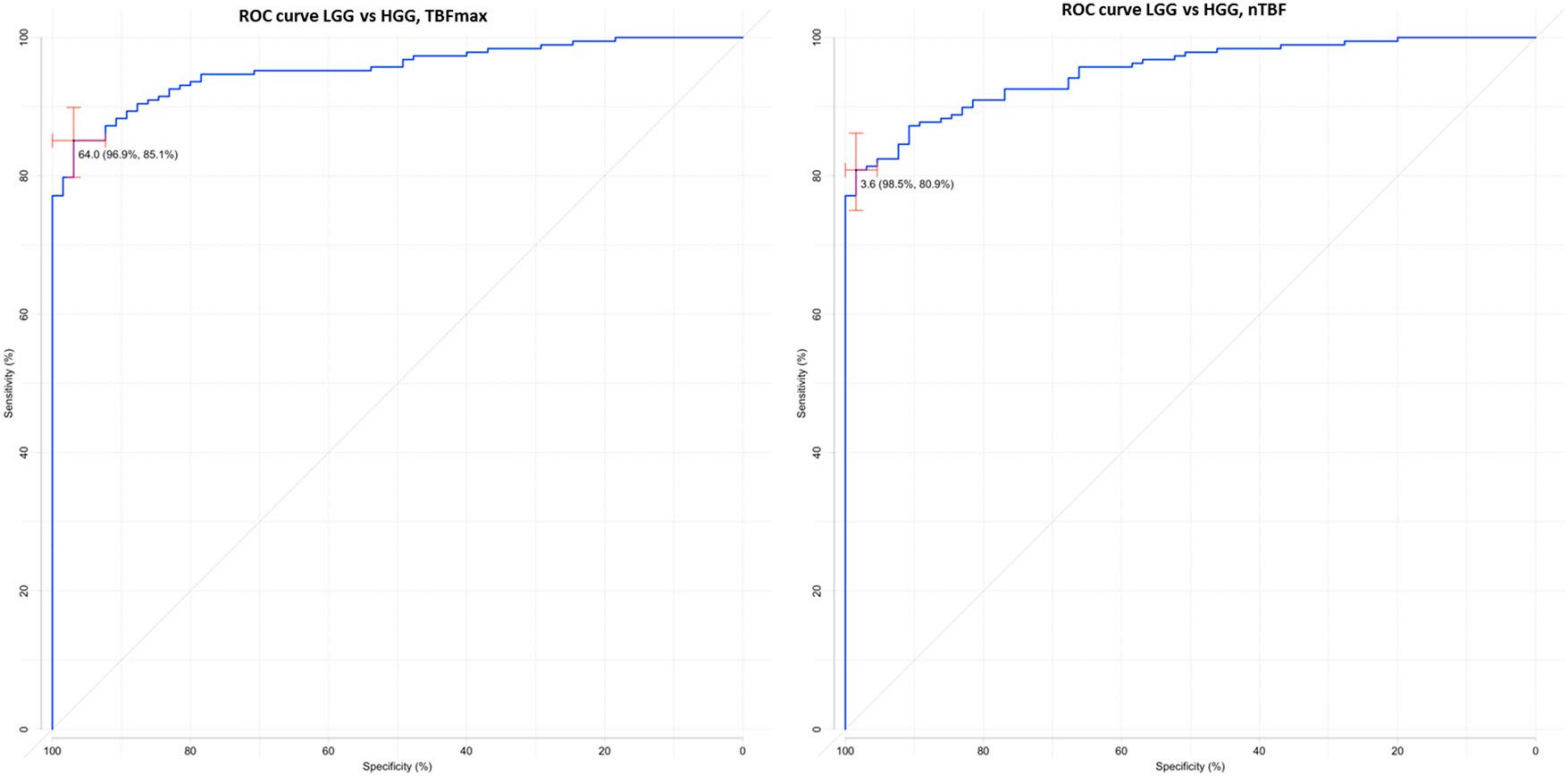
ROC-curve. Comparisons of maxTBF (**a**) and nTBF (**b**) in HGGs and LGGs.

We next calculated the specificity and sensitivity of pCASL-perfusion method in the differential diagnosis between LGG and HGG groups. Data are summarized in Table 3 and Fig. 1.

**Table 3:** ROC-analysis of maxTBF and nTBF in differential diagnosis of brain gliomas.

		maxTBF	nTBF
HGGs and LGGs	AUC	0.954	0.951
	Cutoff	64.0 ml/100 g/min	3.6
	Specificity	96.9%	98.5%
	Sensitivity	85.1%	80.9%
Grade II and Grade III tumors	AUC	0.923	0.921
	Cutoff	44.8 ml/100 g/min	2.7
	Specificity	83.6%	90.9%
	Sensitivity	88.2%	77.9%
Grade III and Grade IV tumors	AUC	0.671	0.656
	Cutoff	103.7 ml/100 g/min	4.7
	Specificity	54.4%	42.6%
	Sensitivity	76.7%	84.2%
Anaplastic astrocytomas and glioblastomas	AUC	0.677	0.664
	Cutoff	114.4 ml/100 g/min	7.0
	Specificity	60.9%	60.9%
	Sensitivity	72.5%	62.5%

pCASL-perfusion method showed both high sensitivity and specificity in distinguishing HGG from LGG. Threshold was determined as 64 ml/100 g/min for maximum TBF and 3.6 for normalized TBF for AUC > 0.95 (Fig. 1).

Absolute maxTBF and nTBF values in low grade gliomas (mTBF = 31.7 ± 14.5 ml/100 g/min; nTBF = 1.8 ± 0.8) were more homogeneous than in high-grade gliomas (TBF = 153.6 ± 93.1 ml/100 g/min; nTBF = 8.5 ± 5.5) (*p* < 0.001). Standard deviation of TBF in the group of LGG turned out to be smaller than in HGG group.

Neither maxTBF nor nTBF values differed between two groups of gliomas, grade I (*n* = 10) and grade II (*n* = 55) (*p* > 0.05). In contrast, we observed significant differences for both maxTBF and nTBF values between grade III (*n* = 68) and grade IV (*n* = 120) gliomas (*p* < 0.001).

**Figure 2:**
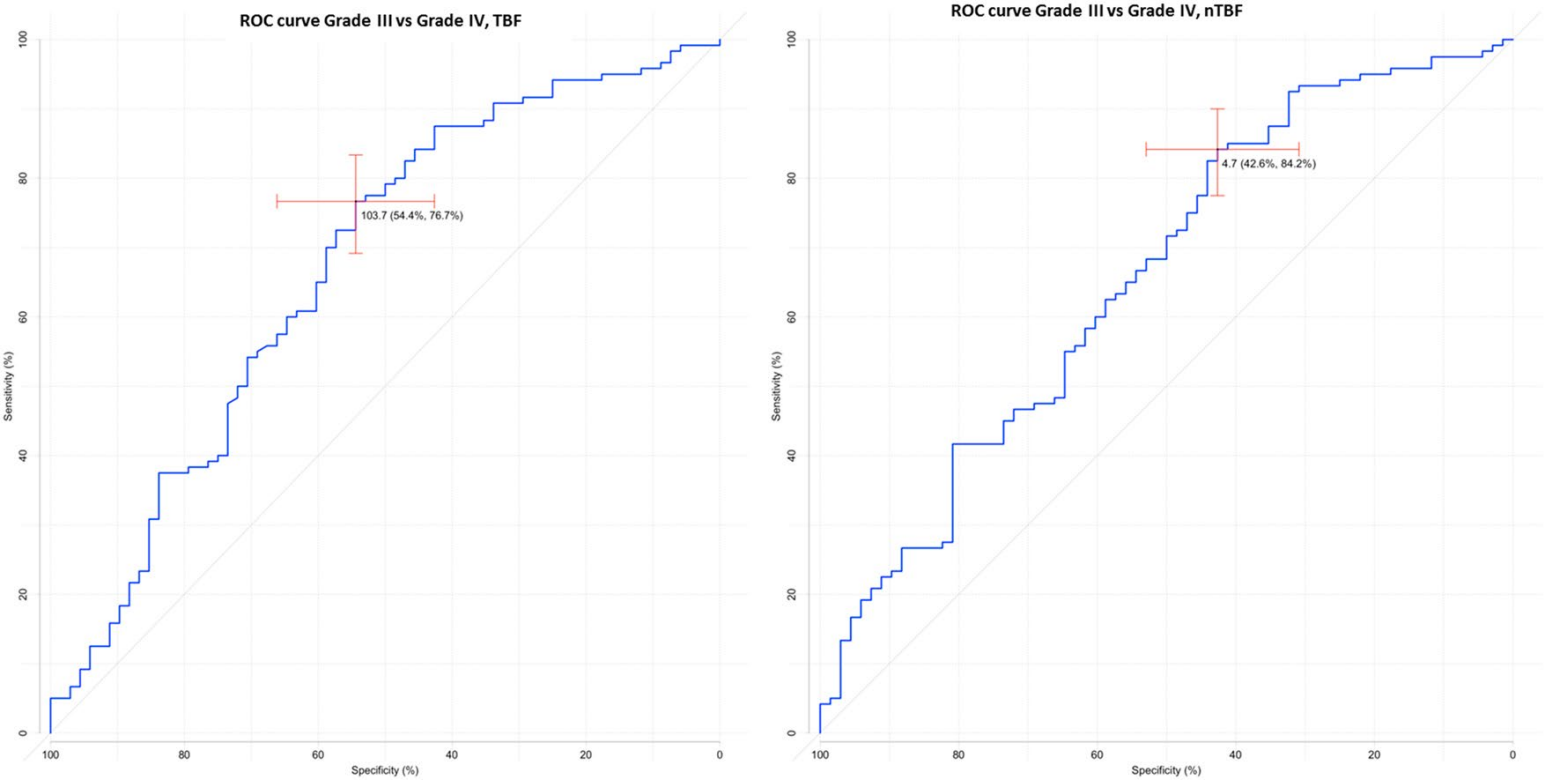
ROC-curve. Comparisons of maxTBF (**a**) and nTBF (**b**) in Grade III and Grade IV tumors.

MaxTBF values were significantly higher in the glioblastomas (grade IV) as compared to the grade III gliomas (anaplastic astrocytomas (*n* = 44), anaplastic oligodendrogliomas (*n* = 22), and anaplastic pleomorphic xantoastrocytomas (*n* = 2)). MaxTBF values for glioblastomas were 171.1 ± 93.3 ml/100 g/min, maxTBF values for grade III gliomas were 122.9 ± 85.1 ml/100 g/min (*p*-values < 0.001). Similarly, nTBF were higher for the glioblastomas: nTBF in glioblastomas—9.5 ± 5.5, nTBF in grade III gliomas—6.8 ± 4.5 (*p*-values < 0.001). However, ROC-analysis showed relatively low specificity and sensitivity of pCASL-perfusion in differentiation of grade III and grade IV gliomas (Table 3, Fig. 2).

Next we compared maxTBF and nTBF between glioblastomas and anaplastic astrocytomas. Both, maxTBF and nTBF values in the anaplastic astrocytomas were significantly lower as compared to the values in the glioblastomas: maxTBF values for anaplastic astrocytomas were 122.9 ± 85.1 ml/100 g/min, maxTBF for glioblastomas 171.1 ± 93.3 ml/100 g/min (*p* < 0.001); nTBF for anaplastic astrocytomas—6.8 ± 4.5, nTBF for glioblastomas—9.5 ± 5.5 (*p* = 0.001).

Exclusion of anaplastic oligodendrogliomas and anaplastic pleomorphic xantoastrocytomas improved neither sensitivity nor specificity when distinguishing grade III from grade IV gliomas using pCASL-perfusion (Table 3).

Comparisons of maxTBF and nTBF within the grade II gliomas (diffuse astrocytomas vs. oligodendrogliomas) and within grade III gliomas (anaplastic astrocytomas vs. anaplastic oligodendrogliomas) showed no significant differences (all *p*-values > 0.05).

Finally, grade III gliomas (*n* = 68) demonstrated significantly higher maxTBF and nTBF values than grade II gliomas (*n* = 55) (both *p* < 0.001). According to ROC-analysis pCASL-perfusion was proved to be highly informative in differential diagnosis of these tumors (Table 3). Exclusion of oligodendrogliomas and anaplastic oligodendrogliomas altered neither sensitivity nor specificity of the method for distinguishing these tumor grades.

### Ethics approval

This retrospective and prospective chart review study involving human participants was in accordance with the ethical standards of the institutional and national research committee and with the 1964 Helsinki Declaration and its later amendments or comparable ethical standards. Approval was granted by the Ethics Committee of "FEDERAL STATE AUTONOMOUS INSTITUTION N.N. BURDENKO NATIONAL MEDICAL RESEARCH CENTER OF NEUROSURGERY OF THE MINISTRY OF HEALTH OF THE RUSSIAN FEDERATION" (Date 16.12.16/No. 12/2016).

### Consent to participate

Informed consent was obtained from all individual participants included in the study. Informed consent was also obtained from the parent and/or legal guardian of the minors who participated in the study.

### Consent for publication

Patients signed informed consent regarding publishing their data and photographs.

## Discussion

This study demonstrated the effectiveness of pCASL in glioma grades differentiation. In our study we used absolute TBF and normalized TBF maximal tumor blood flow. Both metrics showed high sensitivity and specificity in ROC-analysis in differentiating between low- and high-grade gliomas (for maxTBF 85.1% and 96.9%, and for nTBF 80.9% and 98.5% respectively).

We revealed significant differences in absolute and normalized TBF for grade III and grade IV gliomas, although low sensitivity and specificity of these TBF parameters did not allow differentiating between them. Importantly, excluding anaplastic oligodendrogliomas had no effect either on sensitivity or specificity.

Only few studies with relatively small sample sizes compared TBF values using pCASL in grade III and grade IV glioma groups^23–34^. Zeng et al.^28^ revealed significant differences in TBF and nTBF values for these groups of patients (gliomas, grade III *n* = 17, grade IV *n* = 28), but no further analysis of sensitivity and specificity has been performed. Wang et al.^30^ did not find any significant differences for TBF and nTBF in gliomas, grade III, *n* = 13, grade IV, *n* = 24). Our study has the largest sample size of glioma grade III (*n* = 68) and grade IV (*n* = 120) studied, thus increasing the reliability of the obtained results.

Most of the studies also discovered significant differences in blood flow between HGG and LGG^24–34^. The results of our study agree with Xiao et al.^27^, who used a similar approach. They placed several 28–32 mm^2^ ROI within the astrocytic tumors, defined ROIs with the maximal TBF, and normalized absolute TBF values to the cerebellar white matter. Xiao et al. used post-labeling delay of 1525 ms, and they found the cut-off of 2.34 in nTBF to be informative in differentiating low vs. high grade gliomas, with achieved specificity of 84.2% and sensitivity of 91.7%, and AUC of 0.92^27^.

In contrast, Brendle et al.^23^ did not find any significant differences in TBF between LGG and HGG. We suggest that these null-results could be explained by the TBF measurement method applied in this study. The authors used VOIs for TBF measurements which covered the whole glioma volume, and might have averaged out low- and high blood flow values from different areas in the same tumor.

Zeng et al.^28^ used ROIs that included whole area of glioma on the axial slice with the highest TBF. Authors revealed significant differences between the TBF values in HGG and LGG. The AUC was 0.863 with specificity and sensitivity of 84.6% and 82.2% respectively, which is lower than in our study. In contrast to our results, these authors established that excluding the oligodendrogliomas improved the specificity and sensitivity of TBF measurement. In our opinion this difference in the results could be explained by smaller sample size used by Zeng et al., since the larger the sample is the less influence is noted by inclusion/exclusion factors.

All studies with small-size ROI and VOI demonstrated higher sensitivity and specificity in distinguishing of low- and high-grade gliomas. According to a meta-analysis performed by Alsaedi et al.^33^, maxTBF has been proven to be more informative than mean TBF in differentiating between cerebral glioma grades. We have acquired the same results in our study as well.

Overall, our results are in agreement with the previous studies that used small-size ROIs, but we found higher sensitivity, specificity and AUC for pCASL TBF values in differentiating between LGG and HGG. We found a larger difference in maxTBF values between LGG and HGG driven by both lower values for LGG and higher values for HGG as compared to previous reports^23–34^. Importantly, our imaging parameters including post-labeling delay were compatible to those used in previous studies^22–34^. These observed differences in results could be explained by variability in methods of the ROI selection.

In our study we compared maxTBF and nTBF between grade II and III gliomas and established pCASL to be an efficient method for distinguishing between these two groups of tumors. Exclusion of oligodendrogliomas had no effect on specificity or sensitivity of this analysis.

In contrast to previous findings by Zeng et al.^28^, we found no difference in TBF values between diffuse astrocytomas and oligodendrogliomas (grade II) as well as between anaplastic astrocytomas and anaplastic oligodendrogliomas (grade III). In this previous work by Zeng et al.^28^ exclusion of oligodendrogliomas and anaplastic oligodendrogliomas resulted in the average decrease of TBF values in the remaining group of gliomas. The authors also obtained significant differences in TBF between diffuse (grade II) and anaplastic astrocytomas (grade III). This controversy could be explained by ROI selection method: we selected small ROIs in the highest tumor blood flow areas, whereas Zeng et al.^28^ delineated the whole tumor volume on the slice with the maximal TBF.

Accurate histopathological analysis performed by Guo et al.^35^ revealed that oligodendrogliomas are characterized by overall higher microvascular density within the whole tumor volume relative to the other gliomas^35^. Therefore, inclusion of the whole tumor area on the slice into ROIs, used to measure the TBF, might result in the inflated values of the TBF in oligodendrogliomas. Overall, our results suggest that application of small targeted ROIs to measure TBF by ASL-perfusion could be useful in differentiating gliomas of different grades. In addition to that, we calculated slightly higher AUC for max TBF so it could be better to use maximal values of TBF than normalized values. Nevertheless, our normalized results could be useful for studies performed on other scanners (for example 1.5 T scanners) or with other ASL-techniques (for example PASL).

While our results are promising, the study has several limitations. First, our study has a retrospective design. Second, we did not study the association between IDH-1-mutation and tumor blood flow, which is the goal for our further study. However, it is known that IDH mutation might affect TBF value. Finally, our groups included both adult and pediatric patients who are known to have different blood flow properties and need to be studied separately in the future.

## Conclusion

3D pCASL perfusion method with small ROIs for maxTBF and maxTBF normalization showed high sensitivity and specificity in distinguishing high- from low-grade gliomas, as well as grade II from grade III gliomas. This study demonstrated that 3D pCASL- perfusion is an effective diagnostic tool for preoperative differentiation of glioma grades.

## Data Availability

The datasets generated during and/or analysed during the current study are available from the corresponding author on reasonable request.
